# High-Throughput Fabrication of Triangular Nanogap Arrays for Surface-Enhanced Raman Spectroscopy

**DOI:** 10.1021/acsnano.1c09930

**Published:** 2022-04-05

**Authors:** Sihai Luo, Andrea Mancini, Feng Wang, Junyang Liu, Stefan A. Maier, John C. de Mello

**Affiliations:** † Department of Chemistry, Norwegian University of Science and Technology (NTNU), 7491 Trondheim, Norway; ‡ Chair in Hybrid Nanosystems, Nanoinstitute Munich, Faculty of Physics, Ludwig-Maximilians-Universität München, Königinstrasse 10, 80539 München, Germany; § Department of Structural Engineering, Norwegian University of Science and Technology (NTNU), Trondheim 7491, Norway; ∥ College of Chemistry and Chemical Engineering, Xiamen University, Xiamen 361005, China; ⊥ Blackett Laboratory, Imperial College London, Prince Consort Road, London SW7 2BZ, United Kingdom

**Keywords:** nanogaps, lithography, self-assembly, surface-enhanced Raman spectroscopy, nanofabrication

## Abstract

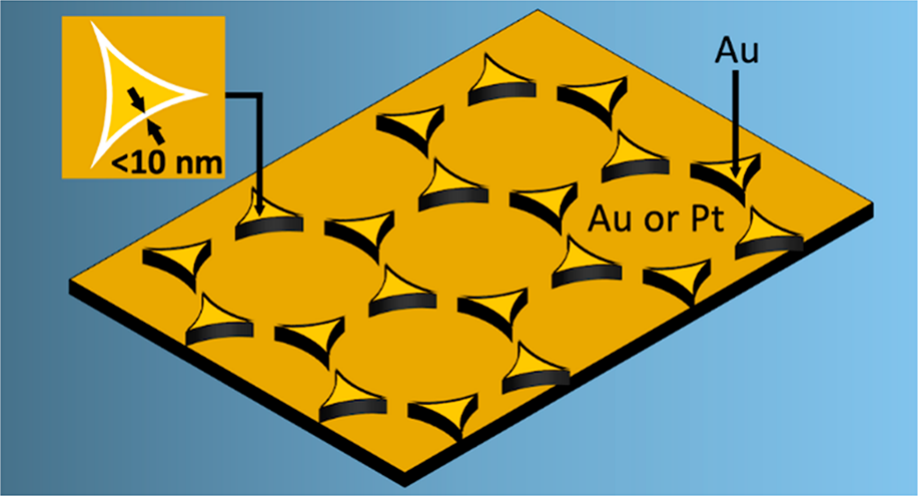

Squeezing light into nanometer-sized metallic nanogaps can generate extremely high near-field intensities, resulting in dramatically enhanced absorption, emission, and Raman scattering of target molecules embedded within the gaps. However, the scarcity of low-cost, high-throughput, and reproducible nanogap fabrication methods offering precise control over the gap size is a continuing obstacle to practical applications. Using a combination of molecular self-assembly, colloidal nanosphere lithography, and physical peeling, we report here a high-throughput method for fabricating large-area arrays of triangular nanogaps that allow the gap width to be tuned from ∼10 to ∼3 nm. The nanogap arrays function as high-performance substrates for surface-enhanced Raman spectroscopy (SERS), with measured enhancement factors as high as 10^8^ relative to a thin gold film. Using the nanogap arrays, methylene blue dye molecules can be detected at concentrations as low as 1 pM, while adenine biomolecules can be detected down to 100 pM. We further show that it is possible to achieve sensitive SERS detection on binary-metal nanogap arrays containing gold and platinum, potentially extending SERS detection to the investigation of reactive species at platinum-based catalytic and electrochemical surfaces.

## Introduction

Surface-enhanced Raman spectroscopy (SERS) has been widely used as a sensitive detection method for chemical analysis, biomedical sensing, and catalysis.^1−8^ The metallic substrate plays a critical role in SERS detection, with nanoscale features on the metal surface acting as “hot-spots” of high electromagnetic (EM) field, where the Raman response of the adsorbed molecules is greatly amplified relative to molecules on a flat metal film, allowing for extremely sensitive detection.^9^ Various types of metallic films have been used for SERS applications, including deliberately roughened or wrinkled films, and lithographically patterned films containing 2D periodic arrays of nanostructured features such as holes, stars, or gaps that serve as EM hot-spots.^10−14^ Array-based substrates are particularly attractive for SERS applications since the periodicity of the engineered hot-spots provides spatially uniform enhancement factors, which is a prerequisite for quantitative SERS analysis. However, the difficulty of patterning periodic arrays over large areas has severely limited their application.

Various nanofabrication methods, such as electron-beam lithography (EBL), extreme-ultraviolet lithography (EUVL),^15−18^ focused-ion beam (FIB) milling,^19−21^ breaking and cracking methods,^22−25^ capillary force-assisted (CFA) lithography,^26,27^ and block copolymer lithography^28,29^ have been used to pattern ordered metallic arrays with SERS-active nanostructured motifs. However, EBL, EUVL, and FIB methods are too costly and time-consuming for fabricating dense nanostructure arrays over wide areas. Breaking and cracking methods offer access to extremely narrow gaps, but the physical structuring of the break junctions prior to the breaking step is typically carried out by EBL or FIB milling so they too suffer from limitations of throughput. CFA lithography and block copolymer lithography meanwhile are restricted to very specific geometries, e.g., pillars and concentric rings, respectively. In addition, another limitation of these methods is that the fabricated nanostructures consist of only a single material, and consequently, they cannot be used to pattern binary nanostructures, which in some circumstances can offer improved Raman sensitivity.^28,30−33^ Therefore, there is an ongoing need for a rapid, inexpensive, and reproducible method for patterning nanostructures with gap widths of 10 nm and below.

Recently, Oh et al. demonstrated an alternative fabrication method,^13,34^*atomic layer lithography*, that overcomes many of the limitations identified above, allowing for the controlled fabrication of dense nanogap arrays over large areas with (optionally) dissimilar metals. The method uses an ultrathin layer of a sacrificial metal oxide grown by atomic layer deposition to establish a nanogap separation between two sequentially deposited metal layers, with the oxide layer then being removed by wet etching to leave an open nanogap between the two metals. In an initial report, using standard lithography techniques such as EBL or FIB to pattern the first metal, they fabricated dense arrays of vertically oriented nanogaps with ∼1 μm pitch and areas of up to 100 μm^2^.^34^ They subsequently eliminated the need for FIB patterning and thereby extended the technique to large-area fabrication (1 cm^2^) by depositing the metal layers onto a template of close-packed polystyrene nanospheres, which resulted in the formation of a ring-shaped nanogap around each sphere.^35^ SERS substrates with very high enhancement factors of up to 10^9^ were achieved but, due to the embedded nanospheres, the nanogaps were oriented at a (nonvertical) angle to the substrate.

In this manuscript, we report a simple, low-cost approach for templated, reproducible, and high-throughput parallel fabrication of vertically aligned gold triangular nanogap (TNG) arrays with tunable gap sizes from ∼3 to ∼10 nm. The method combines molecular self-assembly, colloidal nanosphere lithography, and a peeling-based patterning technique known as adhesion lithography.^36^ The triangular nanogap (TNG) arrays can be readily fabricated over length scales of 1 cm or more and exhibit high, spatially uniform enhancement factors of more than 10^8^, permitting molecular detection down to the pM level.

## Results and Discussion

**Figure 1:**
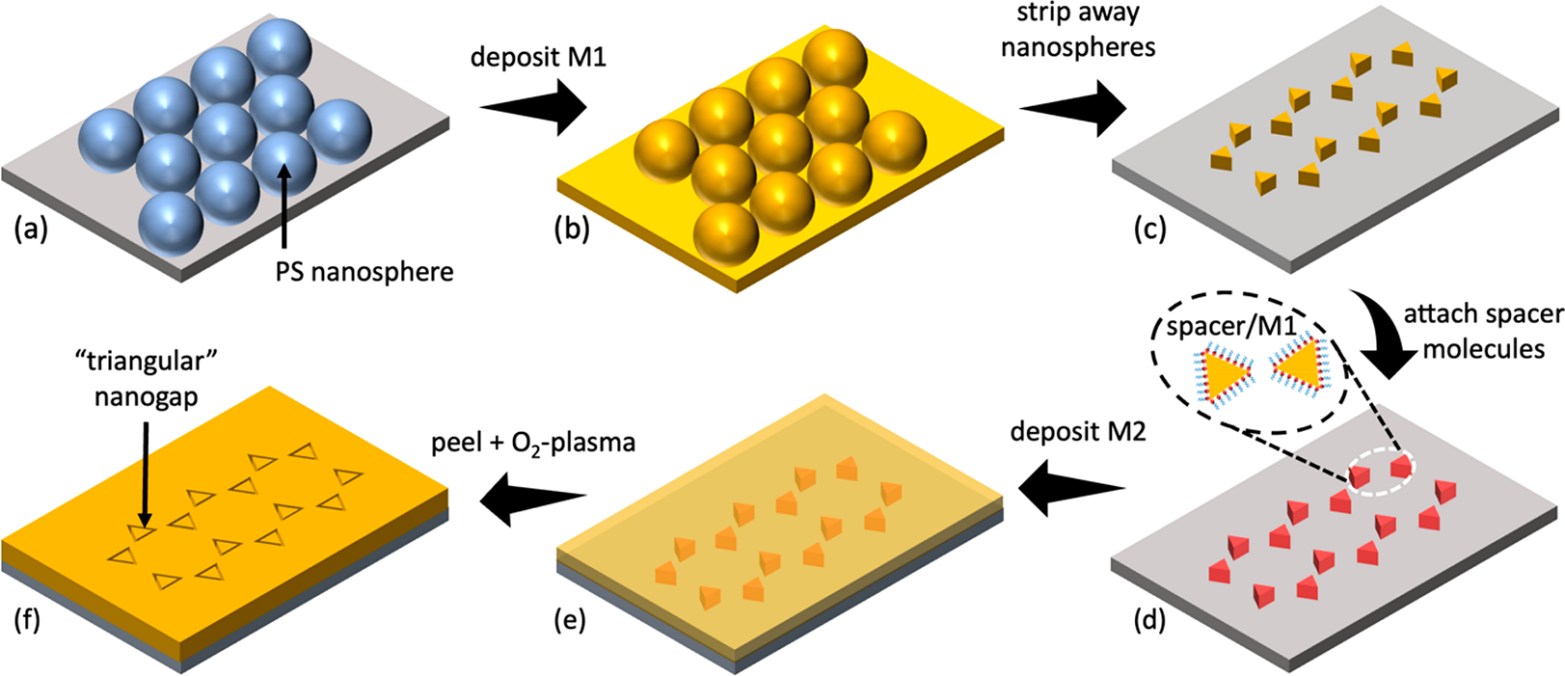
Fabrication procedure for triangular nanogap arrays: first, a monolayer of close-packed polystyrene nanospheres is drop-cast on a substrate and gently treated with an oxygen plasma to reduce surface asperities (a); second, a 50 nm layer of a first metal [M1] is deposited by e-beam deposition onto the nanosphere-coated substrate (b); third, the nanosphere template is removed by tape-stripping, leaving an array of triangular-shaped metal features on the substrate (c); fourth, the metal triangles are conformally coated with a molecular spacer formed from a self-assembled monolayer (SAM) or a self-assembled multilayer (d); fifth, the entire substrate is coated with a 30 nm layer of a second metal [M2] (e); and sixth, an adhesive film is applied to the upper surface of M2 and then stripped away, removing the parts of M2 that lie directly above the first metal. Finally, treatment with an oxygen plasma removes the spacer molecules, leaving M1 and M2 side by side on the substrate with triangular nanoscale gaps between them that are approximately equal in width to the length of the molecular spacer (f).

The fabrication process used to make the TNG arrays is depicted in Figure 1, with further details provided in Figures S1 and S2. First, a close-packed monolayer of 500 nm diameter polystyrene (PS) nanospheres is assembled on a clean glass substrate (Figure 1a) and treated with an oxygen plasma to smooth asperities on the surface of the nanospheres. A thin metal film (M1) of thickness 50 nm is then deposited onto the substrate through the triangle-like voids in the nanosphere array (Figure 1b), and the etched nanospheres are removed by tape-stripping, leaving a periodic 2D array of approximately triangular nanoscale features (Figure 1c). Metalophilic spacer molecules are conformally attached to the top and vertical-sidewall surfaces of the patterned metal by immersing the substrate in a solution of the spacer molecules, followed by thorough rinsing to remove unattached spacer molecules (Figure 1d). A second (thinner) metal (M2) of thickness 30 nm is then evaporated over the full area of the substrate (Figure 1e). Next, an adhesive polymer film is applied over the surface of M2 and then peeled away, causing the parts of M2 that lie above the spacer molecules to be removed.^36−38^ Hence, the two metals M1 and M2 are left sitting side-by-side on the substrate in a complementary arrangement, separated by the SAM. Lastly, O_2_ plasma cleaning is used to remove the SAM molecules, yielding air-filled triangular nanogaps between M1 and M2 (Figure 1f).

To achieve a clean, narrow gap between the two metals, it is important for M2 to be substantially thinner than M1, as this prevents M2 from conformally coating M1. The height difference forces M2 to split along the edge profile of M1 during the e-beam deposition step, vertically separating the unwanted parts of M2 that lie above M1 from the retained parts of M2 that lie directly above the substrate. This in turn allows the unwanted parts of M2 to be removed cleanly during the peeling step without any tearing of M2 or damage to M1, leading to a sharp interface between the two metals.^38^

In common with nanogap arrays fabricated by atomic layer lithography, the arrays reported here are only exposed to the environment after the peeling step. Hence, it is possible to keep the peeling layer in place until the nanogap array is ready to be used. Chen et al. have previously made use of the ability to defer the peeling step in surface-enhanced infrared absorption (SEIRA) sensing, carrying out the peeling step immediately before the sensing experiments were started to minimize surface contamination of the plasmonic array.^39^ The same deferred peeling approach may be applied to the TNG arrays reported here, with it being necessary only to subject the freshly uncovered arrays to a brief oxygen plasma treatment before use.

For most of the work reported here, we used gold for the two metal layers (M1 and M2), although we show at the end of this paper that the technique may also be used to fabricate TNGs between dissimilar metals. The spacers are formed from thiolated self-assembled monolayer (SAM) molecules that attach strongly to gold via the thiol group. For the narrowest gap size, we use a single layer of an alkyl-functionalized thiol SAM (octadecanethiol, ODT), while for wider gaps we use a multilayer spacer formed from a chain of *N* – 1 carboxylic-acid-functionalized SAM molecules (mercapto-hexadecanoic acid, MHDA) and one alkyl functionalized SAM molecule (ODT). The multilayer spacers (commonly referred to as molecular rulers^38,40^) are formed in a stepwise manner by alternately immersing the gold-coated substrate in an ethanolic solution of the SAM molecules and copper perchlorate (see Figure S1 and S2). The first layer of MHDA molecules conformally coats the prepatterned layer of M1 (Au), with the carboxylic acid groups facing outward. Immersion in copper perchlorate then causes copper(II) ions to coordinate with the carboxylic acid groups of MHDA, forming an atomically thin copper layer that serves as a linker upon which a second MHDA layer may be conformally attached. With each cycle, an additional SAM is added to the multilayer, increasing the layer thickness by about 2 nm until the desired thickness is obtained. To minimize adhesion of the spacer layer to M2, ODT is used instead of MHDA for the uppermost layer of the multilayer, which leaves outwardly facing alkyl groups as the surface onto which M2 is evaporated. For the molecular rulers of “length” *N* = 1 [ODT], *N* = 2 [MHDA/ODT], and *N* = 5 [(MHDA)_4_/ODT] used here, the approximate molecular ruler lengths are 2, 5, and 12 nm, respectively.^38,40^

**Figure 2:**
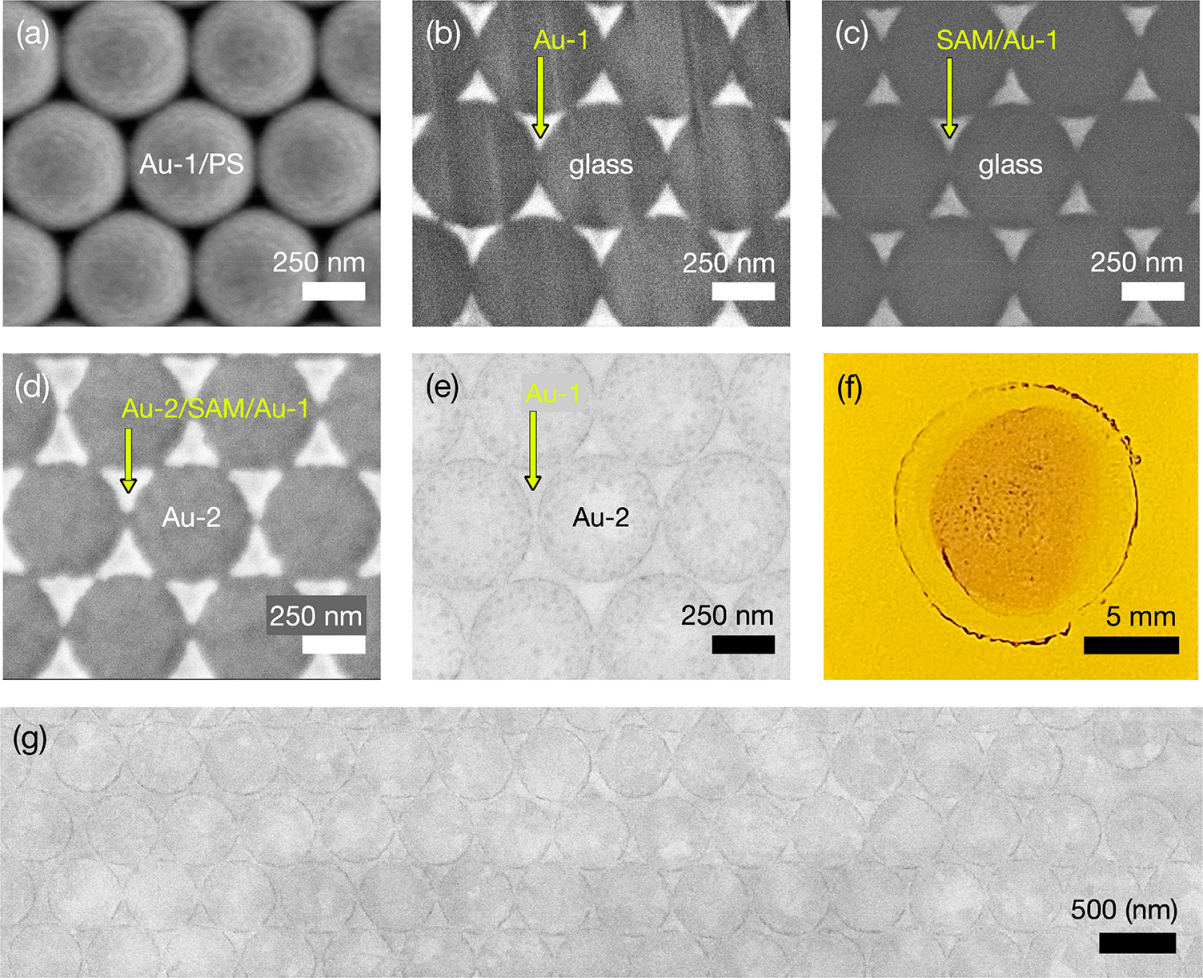
Images of *N* = 1 Au/Au TNG arrays at various stages in the fabrication procedure. (a) SEM image showing a 50 nm gold film (Au-1) on top of a close-packed monolayer of 500 nm diameter polystyrene (PS) spheres on glass. (b) SEM image of the gold-coated glass substrate after removal of the PS nanospheres by tape-stripping. Nanoscale triangles of gold are left behind where gaps previously existed between the nanospheres. (c) SEM image of gold-coated substrate after attachment of ODT spacer molecules. (d) SEM image after deposition of second metal (Au-2). The bright zones are where Au-2 sits directly above (ODT-coated) Au-1, and the dark zones are where Au-2 is in contact with the glass substrate. (e) SEM image of gold-coated substrate after tape-stripping of Au-2 (to remove those parts of Au-2 that lie directly above Au-1) and subsequent oxygen plasma treatment. Au-1 and Au-2 lie side by side on the substrate separated by triangular nanogaps whose width is approximately equal to the length of the molecular spacer. (f) Optical photograph of full nanogap array. (g) Low-magnification SEM image of nanogap array.

Figure 2 panels a–e show illustrative scanning electron micrographs (SEMs) after key processing steps in the fabrication of an *N* = 1 TNG array. Figure 2a shows the situation after depositing the first layer of gold (denoted as Au-1) onto a close-packed monolayer of 500 nm diameter PS nanospheres. The dark regions around the spheres are caused by scattering of electrons in the plane of the monolayer and, contrary to appearance, the spheres typically touch at their equators (as is clear from the image of the final structure shown in Figure 2e). Figure 2b shows the 2D array of triangular gold features left behind on the glass substrate after removal of the PS nanospheres. Figure 2c shows the gold features after they have been conformally coated with ODT molecules. Figure 2d shows the situation after a second layer of gold (Au-2) has been uniformly deposited over the full area of the substrate, with the bright triangular zones corresponding to the “double-thickness” of gold present in the regions where Au-1 and Au-2 overlap and the dark zones corresponding to regions where Au-2 is deposited directly on the substrate. Figure 2e shows the situation after peeling and oxygen plasma treatment: circular discs of Au-2 are present in the areas originally occupied by the PS nanospheres, while triangular patches of Au-1 are present in the triangular regions between the Au-2 discs. A bowtie-shaped nanogap can also be seen where two adjacent triangles have merged due to a gap between adjacent nanospheres (caused by the presence of a slightly undersized nanosphere). Figure 2g shows a lower magnification SEM image of a ∼ 2 μm by ∼8 μm section of the TNG array, while Figure 2f shows a photograph of the entire array. In contrast to conventional nanoscale lithography techniques such as EBL and FIB, using the approach described here, dense arrays of TNGs can be fabricated over cm^2^-sized areas without difficulty (see Figure 2f). A simple geometric calculation shows that there are almost 1 billion (10^9^) TNGs in a typical deposited area of around 1 cm^2^.

**Figure 3:**
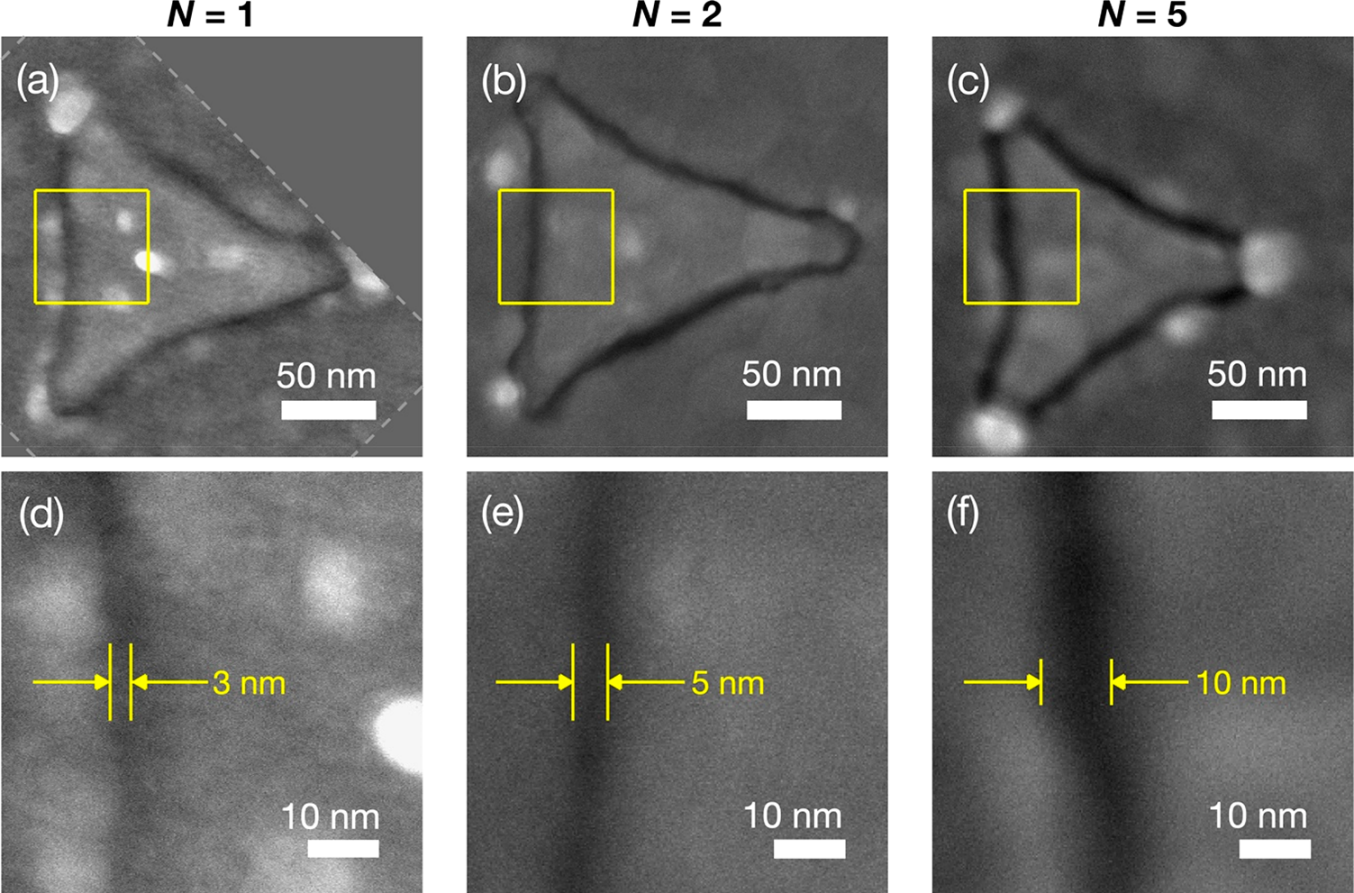
High-resolution SEM images of triangular Au/Au nanogaps. (a)–(c) SEM images showing a single triangular nanogap in an *N* = 1 (a), *N* = 2 (b), and *N* = 5 (c) TNG array. The yellow boxes enclose square regions of length 60 nm. The dotted white lines in (a) indicate the edge of the SEM image, which has been rotated to bring the left edge of the triangle into vertical alignment. (d)–(f) Magnified sections of the SEM images from (a)–(c), showing the yellow boxed regions. The approximate gap widths are 3, 5, and 10 nm for the *N* = 1 (d), *N* = 2 (e), and *N* = 5 (f) TNG arrays.

Figure 3 panels a–c show 225 nm by 225 nm SEM images of typical triangular nanogaps formed using molecular rulers of length *N* = 1 (ODT), *N* = 2 (MHDA/ODT), and *N* = 5 ([MHDA]_4_/ODT), with Au-1 filling the interior of the triangles and Au-2 surrounding the triangles. The thin black “lines” separating the two regions correspond to the air-filled nanogaps left behind after removal of the molecular spacers. Figure 3 panels d–f show representative high-resolution SEM images of the gap regions in the three arrays. The images indicate gap widths of ∼3, ∼5, and ∼10 nm, which correspond closely to the 2, 5, and 12 nm lengths of the three molecular rulers. Lower magnification SEM images of the TNG arrays are provided in Figure S3.

**Figure 4:**
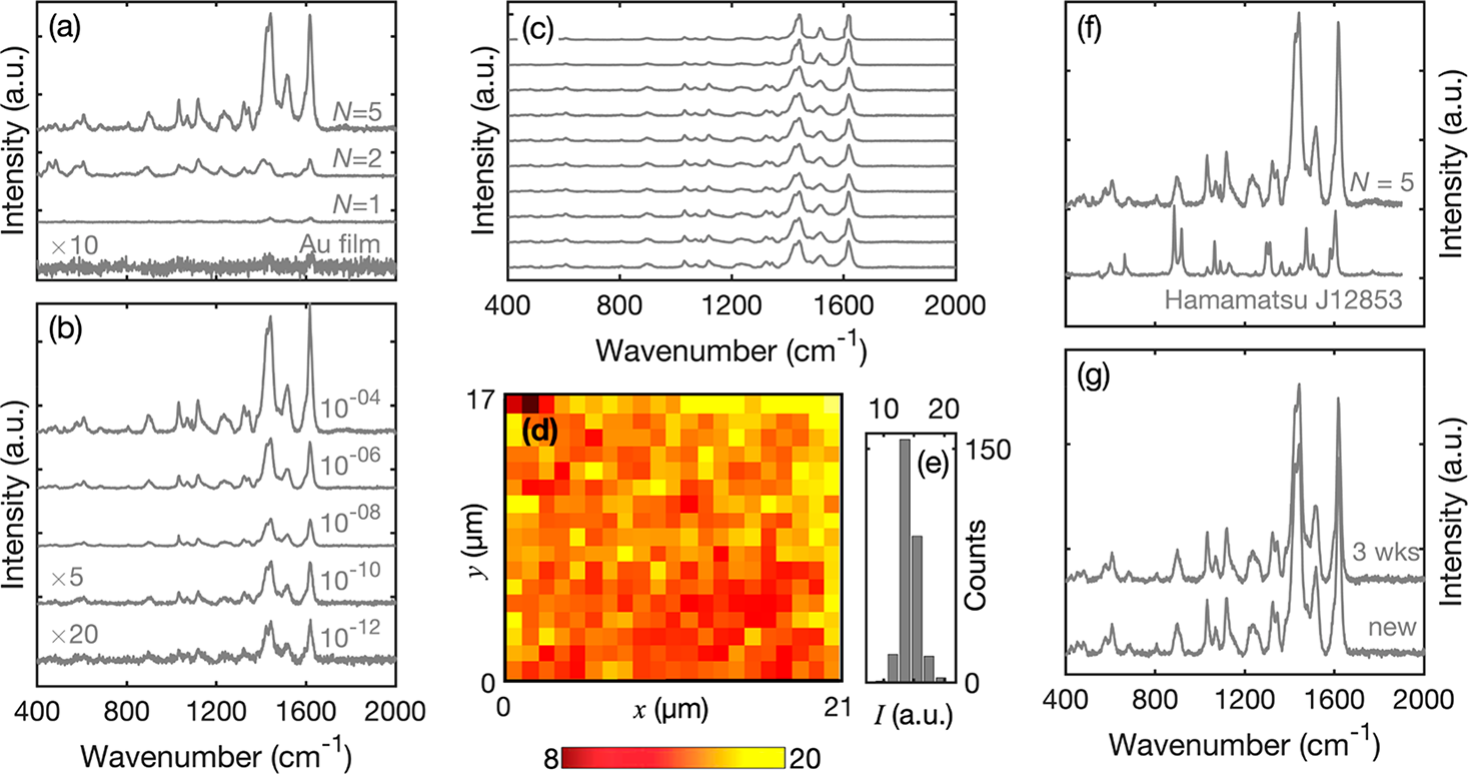
Surface-enhanced Raman scattering from Au/Au TNG arrays. (a) Experimentally determined Raman scattering spectra for methylene blue (MB) drop-cast from a 10^–4^ M solution onto Au/Au TNG arrays fabricated using 500 nm diameter PS nanospheres and molecular spacers of length *N* = 1, 2, and 5. Also shown for comparison is a Raman scattering spectrum for 10^–4^ M MB drop-cast onto a thin gold film. Spectra were obtained under equivalent conditions with a 785 nm excitation wavelength; see the Experimental Section. The spectrum for the thin gold film has been multiplied by a factor of 10 for clarity. (b) Raman scattering spectra for MB drop-cast onto *N* = 5 Au/Au TNG arrays from MB dye solutions of varying concentration. Spectra were obtained under equivalent conditions with a 785 nm excitation wavelength. The spectra for 10^–10^ and 10^–12^ M MB have been multiplied by respective factors of 5 and 20 for clarity. (c) Raman scattering spectra for MB drop-cast from a 10^–4^ M solution onto an *N* = 5 TNG array. Spectra were obtained at ten arbitrary locations under fixed 785 nm illumination. (d) Raman intensity map, showing the point-by-point Raman intensity over a 17 μm × 21 μm region of the *N* = 5 TNG array, using a step size of 1 μm and a laser spot diameter of 1 μm. (e) Histogram showing the distribution of Raman intensities at 1616 cm^–1^ extracted from the data in (d). (f) Raman scattering spectra for MB drop-cast from a 10^–4^ M solution onto an *N* = 5 Au/Au TNG array of approximate area 1 cm^2^ and a commercial SERS substrate (Hamamatsu J12853) of area 0.07 cm^2^. (g) Raman scattering spectra for MB drop-cast from a 10^–4^ M solution onto a freshly fabricated *N* = 5 Au/Au TNG array and an equivalent three-week-old array.

**Figure 5:**
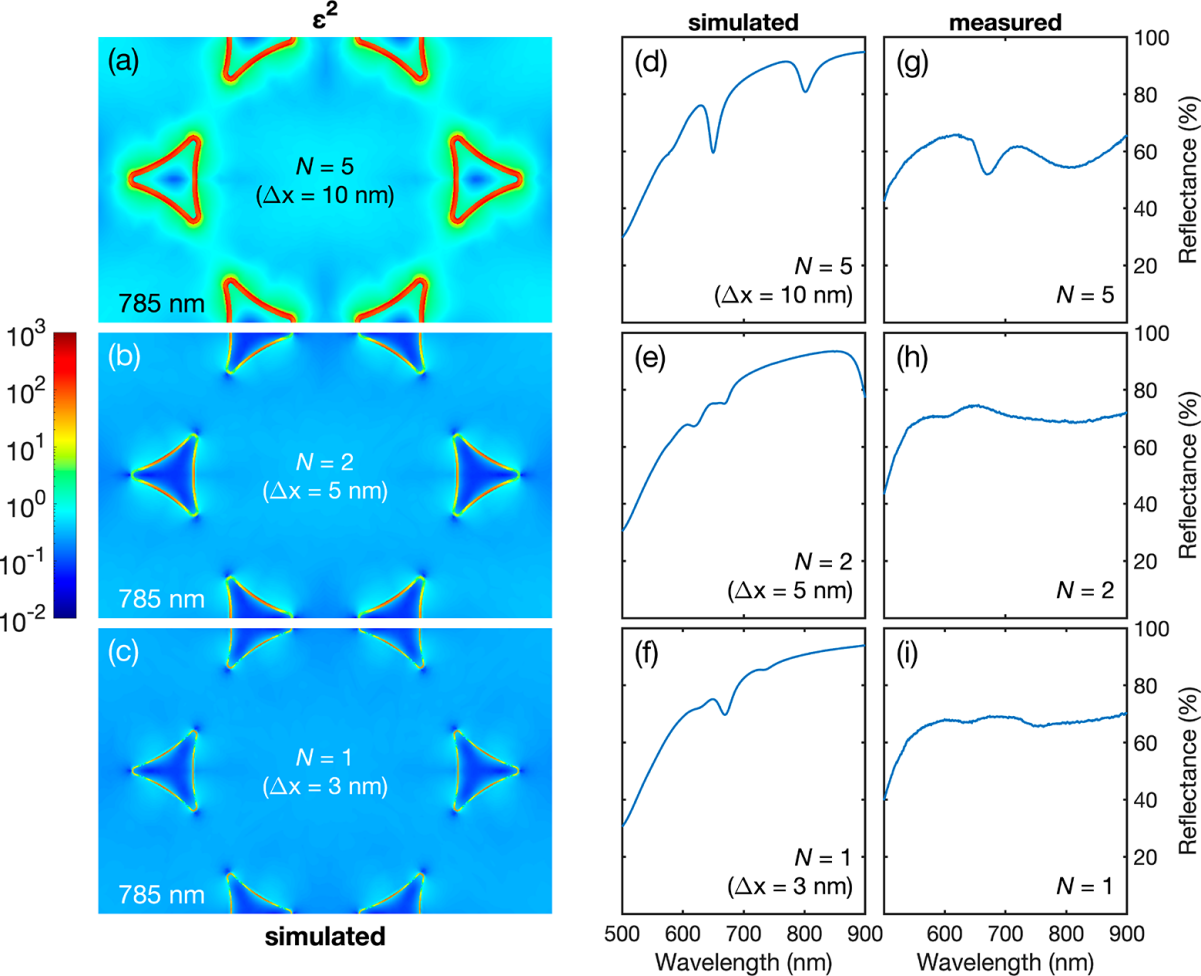
Simulated field-enhancement maps and simulated and experimental reflectance spectra for Au/Au TNG arrays. (a)–(c) Simulated plots showing the square of the field enhancement  at a height z* = 30 nm above the glass substrate (i.e., coincident with the top surface of Au-2) for gap widths of 3 nm (*N* = 1), 5 nm (*N* = 2), and 10 nm (*N* = 5), assuming an unpolarized plane wave illumination at 785 nm. (d)–(f) Simulated reflectance spectra for gap widths of 3 nm (*N* = 1), 5 nm (*N* = 2), and 10 nm (*N* = 5), assuming an unpolarized, monochromatic plane-wave illumination in the range 500–900 nm. (g)–(i) Experimentally determined reflectance spectra for *N* = 1, *N* = 2, and *N* = 5 TNG arrays, using an unpolarized monochromatic, plane-wave illumination in the range 500–900 nm.

Under near-resonant illumination conditions, periodic arrays of metallic nanogaps can generate tremendous electromagnetic field enhancements due to extreme localization of the incident light, which in turn can cause nearby molecules to display a range of surface-enhanced optical properties such as increased absorption, Raman scattering, second-harmonic generation, and chiroptical behavior.^9,41−44^ To test the performance of the TNG arrays as SERS substrates, a 10^–4^ M solution of the widely used Raman probe methylene blue (MB) was drop-cast onto the three (*N* = 1, 2 and 5) Au/Au TNG arrays, and Raman spectra were recorded under equivalent conditions at a probe wavelength of 785 nm (see Experimental Section). The spectra are shown in Figure 4a, together with a spectrum for a thin gold film of thickness 30 nm obtained under equivalent conditions. The SERS intensities obtained using the TNG arrays are substantially higher than for the thin gold film and increase progressively as the spacer length *N* is increased from 1 to 5, i.e., from ∼3 nm to ∼10 nm. This behavior differs from results we have previously reported for ring-shaped nanogaps, where increasing the spacer length from 1 to 5 led to a progressive *decrease* in the SERS activity.^38^ The optimum gap width depends on the geometry of nanogaps, and narrower gaps do not always lead to stronger SERS responses. Using wedge-shaped nanogaps for instance, Chen et al. found that a ∼ 2 nm gap width gave a stronger SERS response than a ∼ 1 nm gap width.^45^ For the triangular nanogap geometry investigated here, simulations indicate the increase in SERS activity as *N* is increased from 1 to 5 is attributable to a progressive increase in the field-enhancement within the gap as the gap width increases from 3 to 10 nm (see Figure 5 and associated discussion).

Figure 4b shows Raman spectra obtained using the *N* = 5 TNG array for a series of MB solutions, ranging in concentration from 10^–4^ to 10^–12^ M. A gradual reduction in the Raman intensity was observed with decreasing dye concentration, but even at 1 pM it was still possible to discern the characteristic peaks of MB. An approximately logarithmic relationship was observed between the Raman signal and the dye concentration. Comparing to the thin gold film, we deduce an enhancement factor of more than 10^8^ (see Figure S4), which compares favorably with other recently reported SERS substrates (see Table S1). We note that the 785 nm excitation wavelength used here is far beyond the 700 nm absorption onset (and the 670 nm absorption peak) of methylene blue, so we are working in the nonresonant Raman regime where chemical resonance effects are negligible. Figure S5 shows a comparison of the Raman response under 532, 633, and 785 nm excitation. For both 633 and 785 nm illumination, there is a strong increase in Raman signal as *N* is raised from 1 to 5, while at 532 nm the Raman signal is extremely weak for all three values of *N*.

Figure 4c shows Raman spectra measured at ten arbitrary spots on the array and indicates a high degree of consistency in the strength of the Raman signal from one location to the next. The homogeneity of the SERS signals on a microscopic length scale was determined by point-by-point Raman mapping over an area of 17 μm × 21 μm, using a step size of 1 μm and a laser spot of diameter 1 μm. The relative standard deviation (RSD) of the signal was around 9.7% (see Figure 4d,e), which is better than the acceptable 20% threshold for quantitative applications.^12^Figure 4f shows a comparison between the signal obtained from a (∼1 cm^2^) *N* = 5 TNG array and a commercial SERS substrate from Hamamatsu (Product Number J12853) with an active area of 0.07 cm^2^; the performance of the TNG array is at least as good as the commercial substrate.

Beyond the high enhancement factors, the TNG arrays also show excellent environmental stability, as can be seen for instance from Figure 4g, which shows virtually identical SERS signals from a freshly fabricated array and one that has been stored under ambient conditions for 3 weeks. The ability to fabricate the TNG arrays over large areas, combined with their high enhancement factors and excellent environmental stability, make them attractive candidates for many SERS applications.

To understand the strong SERS response at 785 nm and the variation of the SERS signal with the gap width, three-dimensional (finite element method) electromagnetic simulations were carried out for the (uncoated) TNG arrays, assuming a pitch of 500 nm, heights of 50 and 30 nm for Au-1 and Au-2 and gap widths of 3, 5, and 10 nm for the *N* = 1, 2, and 5 TNG arrays. Further details about the geometry and simulation procedure are provided in the Experimental Section and Figures S6 and S7. Figure 5 panels a–c show for the three nanogap arrays under normally incident illumination at 785 nm, the square of the electric field enhancement at a vertical height z* = 30 nm from the *xy* plane of the substrate:
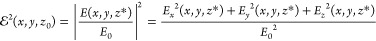
where *E*_*x*_, *E*_*y*_, and *E*_*z*_ are the components of the local electric field vector *E*, and *E*_0_ is the amplitude of the incoming plane-polarized wave. The plane z* = 30 nm coincides with the upper surface of Au-2; it therefore passes across the top of the nanogap separating Au-1 and Au-2 and cuts through the bulk of Au-1, 20 nm below its upper surface. The simulations reveal substantial plasmonic activity under 785 nm illumination, with all three arrays showing enhanced electric field strengths within the nanogaps. A substantial increase in the electric field strength inside the gap is seen as the width of the gap is increased from 2 to 5 to 10 nm, with the mean-squared average electric field enhancement (at a height z* = 30 nm) increasing from 4.4 to 5.0 to 411.8. As the Raman scattering cross-section is approximately proportional to the fourth power of , the large increase in electric field enhancement in going from the *N* = 1 array (3 nm) to the *N* = 5 array (10 nm) accounts qualitatively for the increase in the measured SERS signal.

Figure 5 panels d–f show simulated reflectance spectra for the three arrays. The *N* = 5 array shows two substantial dips in reflectance centered at λ = 650 nm and λ = 802 nm. The dips move to longer wavelengths as the pitch of the array is changed (see Figure S8), suggesting they correspond to collective modes of the TNG array. They are also strongly affected by the curvature at the vertices of the triangles, with higher curvature leading to a blue shift of the dips. Illumination within the wavelength range of the two dips leads to substantial simulated field enhancements inside the gap, consistent with our experimental observation of strong SERS signals when excitation wavelengths of 633 or 785 nm are used. Excitation at 532 nm—the other laser wavelength available to us experimentally, which lies away from the dips—gives only a very weak simulated field enhancement, in agreement with our experimental observation of low Raman signals. The reflectance dips and the corresponding field enhancements are much smaller for the *N* = 2 and *N* = 1 arrays, consistent with the weaker Raman signals measured experimentally. Experimentally measured reflectance spectra are roughly consistent with the simulated spectra, with the *N* = 5 array showing two substantial dips at 670 and 805 nm and the *N* = 2 and *N* = 1 arrays showing much smaller dips. The experimentally observed features are in all cases substantially broader than the simulated features due to inhomogeneity in the shape of the triangular motifs within the arrays. (As noted above, slight differences in the curvature of the nanogaps close to the vertices can lead to substantial differences in the observed reflectance spectrum.)

The TNG arrays may also be applied to the SERS detection of biomolecules. In Figure S9 we show illustrative (experimental) results for adenine, one of the four constituent bases of nucleic acids. Adenine exhibits a characteristic ring-stretching peak in its Raman spectrum close to 731 cm^–1^ that can facilitate the detection of DNA and RNA.^29^Figure S9a shows SERS spectra obtained by drop-casting adenine solutions of varying concentration onto an *N* = 5 TNG array, while Figure S9b shows the Raman signal at 731 cm^–1^ versus concentration. The stretching peak is easily detectable at concentrations as low as 100 pM, confirming the suitability of the TNG arrays for sensitive, label-free detection of biomolecules.

**Figure 6:**
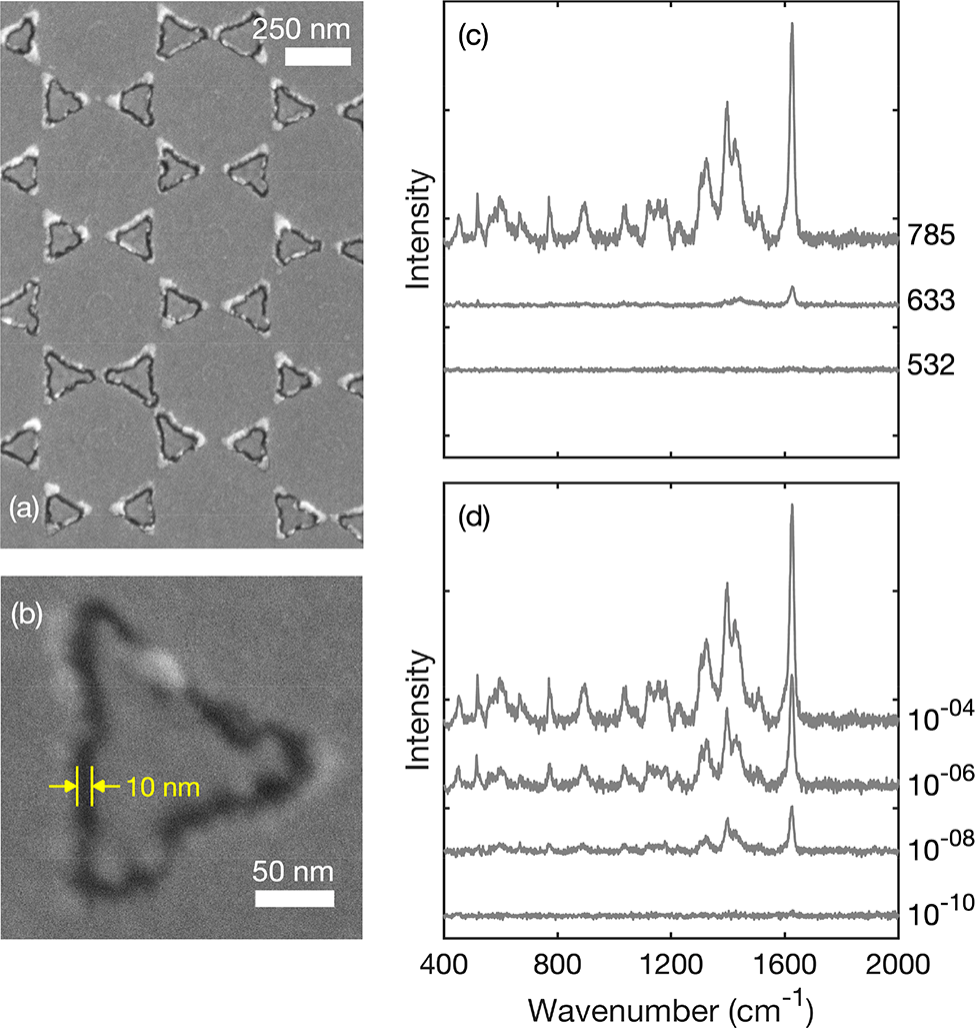
Binary-metal Au/Pt TNG arrays. (a) SEM image of Au/Pt array, fabricated using 500 nm diameter PS nanospheres and molecular spacers of length *N* = 5. The triangles are gold, and the surrounding metal is platinum. (b) High-magnification image of a single *N* = 5 Au/Pt triangular nanogap with an approximate gap width of 10 nm. (c) Raman scattering spectra for methylene blue drop-cast from a 10^–4^ M solution onto an *N* = 5 Au/Pt TNG array under illumination wavelengths of 532, 633, and 785 nm. (d) Experimentally determined Raman scattering spectra for MB drop-cast onto *N* = 5 Au/Pt TNG arrays from MB dye solutions of varying concentration from 10^−4^ M to 10^−10^ M. Spectra were obtained under equivalent conditions with a 785 nm excitation wavelength.

Finally, we note that the patterning procedure may also be applied to the fabrication of asymmetric nanogap structures formed from dissimilar metals, with the gap width again being determined by the width of the spacer layer. In Figure 6a,b we show SEM images and SERS data for a typical TNG array formed from gold (M1) and platinum (M2), using a molecular ruler of length *N* = 5. The approximate gap width of ∼10 nm is similar to that obtained with an equivalent Au/Au TNG array, while the 10 nM detection limit is substantially higher (worse) than the 1 pM limit of the gold-only array due to the weak SERS activity of platinum. (The platinum content of the TNG array is approximately 85% by volume.) We note that there have been many efforts over the past 30 years to apply SERS to Pt-group metals due to their importance in catalysis and electrochemistry (see, e.g., work by the groups of Tian,^46,47^ Perez,^48^ Bartlett,^49^ and Padalkar^50^). The extension of SERS detection to monitoring reactive species at such surfaces would provide a powerful tool for studying electrochemical and catalytic processes. To our knowledge the 100 nM detection limit reported here is the lowest (best) value reported to date for molecules on a Pt-based substrate; see, e.g., ref (50).

## Conclusion

Using a combination of molecular self-assembly, colloidal nanosphere lithography and physical peeling, we have described a simple, high-throughput method for fabricating large-area dense arrays of triangular nanogaps that allows the gap width to be tuned from ∼10 nm to less than 3 nm. The nanogap arrays function as high-performance, spatially uniform substrates for surface-enhanced Raman spectroscopy under 633 and 785 nm illumination, with the SERS activity increasing substantially as the gap width is increased from ∼3 to ∼10 nm. Electromagnetic simulations indicate that the strong SERS activity is due to the excitation of collective plasmonic modes of the array that can result in substantial mean-squared field enhancements of more than 400 at the top of the gap.

Using *N* = 5 (10 nm) gold–gold TNG arrays, MB dye molecules could be detected at concentrations as low as 1 pM under 785 nm illumination. The enhancement factor relative to a thin gold film was more than 10^8^, competitive with many commercial SERS substrates of much smaller size. Using a 10 nm TNG array, we were able to achieve sensitive label-free detection of adenine biomolecules down to 100 pM. Finally, we showed that it is possible to achieve sensitive SERS detection on mixed-metal TNG arrays based on gold and platinum, which raises the prospect of carrying out sensitive SERS analysis of reactive species at catalytic and electrochemical surfaces.

Despite their high SERS activity, the *N* = 5 TNG arrays have not been optimized for SERS applications, and we consider here how the SERS activity might be further increased. Further optimization of the gap width is an obvious first step since the molecular ruler length was limited to *N* = 5 due to yield issues at higher ruler lengths. A further option is to replace gold by silver (which has a much stronger SERS activity), although it would be necessary to replace the oxygen plasma treatment used to remove the molecular spacer by a technique that does not etch silver (e.g., an argon plasma treatment or a compatible reactive ion etch). Simulations indicate that the pitch has a strong effect on the plasmonic behavior of the arrays, and optimization of the pitch (by tuning the nanosphere diameter) may be expected to further improve the SERS activity at the 785 nm excitation wavelength. Other geometric factors such as the metal film thicknesses and the step-height between the two metals (currently fixed at 20 nm) are also likely to influence the SERS activity.

## Experimental Section

### Materials and Chemicals

Deionized water (18.2 MΩ·cm) was obtained from a Millipore filtration system. Glass substrates were cleaned by oxygen plasma before use. Octadecanethiol (ODT, 98%), 16-mercaptohexadecanoic acid (MHDA), Cu(ClO_4_)_2_, polystyrene spheres (500 nm, 10 wt % in water, Product no. 59769, Sigma-Aldrich) and methylene blue (MB, 99%) were purchased from Aldrich. Acetone (99.5% purity) and absolute ethanol (99.5% purity) were used as received from VWR without further purification.

### Fabrication of *N* = 1 Au/Au TNG Arrays

To prepare the nanosphere templates, a glass substrate was sequentially cleaned with acetone, ethanol, and deionized water, dried in a stream of nitrogen, and then subjected to oxygen plasma for 2 min (100 W, O_2_ flow rate: 50 sccm). A circular well of polydimethylsiloxane (PDMS) of diameter 1 cm and height 2 mm was placed in the center of the glass substrate. A 10 wt % suspension of 500 nm diameter PS nanospheres in water was volumetrically diluted by a factor of 3 in ethanol, and loaded into a micropipette. A 0.5 μL droplet of the diluted solution was deposited inside the PDMS well and allowed to dry under ambient conditions, yielding a close-packed monolayer of nanospheres. The nanospheres were subjected to an oxygen plasma treatment for 3 min to smooth their surface without substantially changing their diameter. A 5 nm adhesion layer of titanium, followed by a 45 nm layer of gold (Au-1) was deposited onto the templated substrate by e-beam evaporation (10^–7^ mbar, 2 Ås^–1^). The PS nanospheres were removed using one-sided 3 M Scotch tape, leaving behind a hexagonal array of nanoholes in the gold film. The substrate was then immersed in a 2 mM ethanolic solution of ODT for 24 h, before washing thoroughly in clean ethanol to remove unattached SAM molecules and possible dithiol bridge species. A 30 nm layer of Au (Au-2) was then deposited by e-beam deposition over the full area of the metal-coated substrate (10^–7^ mbar, 2 Ås^–1^). An adhesive film (First Contact Red, Photonic Cleaning Technologies) was drop-cast on top of the substrate, allowed to dry at room temperature, and then peeled away manually, leaving Au-1 and Au-2 side-by-side on the substrate, separated by an ODT monolayer. In the final step, the ODT was removed by O_2_ plasma treatment for 3 min.

### Fabrication of *N* > 1 Au/Au TNG Arrays

Fabrication was carried out using a procedure equivalent to the one used for the *N* = 1 TNG arrays, except the ODT monolayer was replaced by a molecular ruler, i.e., a metal-ligated multilayer. The molecular rulers were prepared according to a modified literature protocol^49^ by first immersing the substrate in a 2 × 10^–3^ M ethanolic solution of 16-MHDA for 12 h to form a densely packed monolayer on top of Au-1. Further layers of MHDA were then added in a stepwise manner by alternately immersing the substrate in a 2 × 10^–3^ M ethanolic solution of copper perchlorate for 15 min and a 2 × 10^–3^ M ethanolic solution of MHDA for 30 min, washing thoroughly in clean ethanol between each process step. In the final step of the multilayer preparation (after Cu(ClO_4_)_2_ treatment), the substrate was immersed in a 2 × 10^–3^ M ethanolic solution of ODT for 24 h, yielding an upper surface of nonreactive alkyl groups in the self-assembled multilayer. From this point onward the fabrication procedure was identical to the *N* = 1 procedure.

### Fabrication of *N* = 5 Au/Pt TNG Arrays

Binary metal Au/Pt arrays were made in a similar manner to equivalent gold-only arrays, except Au-2 was replaced by Pt.

### Imaging

SEM images of the TNG arrays at various stages of fabrication were recorded on a scanning electron microscope (FEI APREO) using an electron-beam voltage of 10 kV and a current of 13 pA.

### Raman Measurements

Raman spectra were obtained on a Renishaw InVia Raman spectrometer with laser excitation wavelengths of 532, 633, and 785 nm. The laser beam was focused onto the sample through a ×50 objective lens, using a fixed power of 0.5 mW and a 10 s acquisition time. To prepare the samples for SERS measurements, the samples were immersed for 4 h in ethanolic solutions of MB ranging from 10^–4^ to 10^–12^ M or aqueous adenine solutions ranging from 10^–4^ to 10^–12^ M. All samples were then rinsed thoroughly with ethanol to remove weakly attached molecules and dried under ambient conditions before carrying out the Raman measurements.

### Electromagnetic Simulations

3D electromagnetic simulations were performed with the commercial software package CST Studio in the frequency domain. To simulate the arrays, periodic boundary conditions were used in the *x* and *y* directions, with a rectangular unit cell of dimensions *P*_*x*_ = 500 nm and *P*_*y*_ = 2·*P*_*x*_ sin(π/3). TNGs were obtained by intersecting slightly overlapping cylinders of radius *r* = 0.51 × *P*_*x*_, with the region complementary to the cylinders forming the triangular structures. The tips of the triangles were blended to have a curvature radius of 5 nm, resulting in simulated reflectance spectra in reasonable agreement with the measured ones. The gap was obtained by creating a shell object from the TNG of size equal to the gap dimension. The maximum mesh step in the gap region was set to 5 nm. The thickness of the gold was set to 50 nm for Au-1 and 30 nm for Au-2 (ignoring the presence of the Ti adhesion layer). A linearly polarized plane wave at normal incidence in combination with Floquet Mode Ports was used to simulate the excitation of the λ = 532, 633, or 785 nm laser wavelengths used for the SERS experiments. The field-enhancement maps and reflectance spectra were obtained by averaging TE and TM polarizations to simulate unpolarized illumination. The arrays were simulated on a glass substrate to match the fabricated structure.

## Supporting Information Available

Further details of the fabrication and simulation procedures, simulated reflectance data, SEM images, and SERS spectra (PDF)
