# Supplementary material for: High-Throughput Fabrication of Triangular Nanogap Arrays for Surface-Enhanced Raman Spectroscopy
Source: ACS Nano. 2022 Apr 5;16(5):7438–47. doi: 10.1021/acsnano.1c09930 (PMC9134500; doi:10.1021/acsnano.1c09930)
Supplement: Supplementary file 1 — nn1c09930_si_001.pdf [file nn1c09930_si_001.pdf]

High-Throughput Fabrication of Triangular Nanogap Arrays for Surface-Enhanced Raman Spectroscopy

Sihai Luo[1], Andrea Mancini[2], Feng Wang[3], Junyang Liu[4], Stefan A. Maier[2,5,6], John C. de Mello[1*]*

[1]*Department of Chemistry, Norwegian University of Science and Technology (NTNU), 7491 Trondheim, Norway*

[2]*Chair in Hybrid Nanosystems, Nanoinstitute Munich, Faculty of Physics, Ludwig-Maximilians-Universität München, Königinstrasse 10, 80539 München, Germany*

[3]*Department of Structural Engineering, Norwegian University of Science and Technology (NTNU), Trondheim, 7491, Norway*

[4]*College of Chemistry and Chemical Engineering, Xiamen University, Xiamen 361005, China*

[5]*School of Physics and Astronomy, Monash University, Clayton Victoria 3800, Australia*

[6]*Blackett Laboratory, Imperial College London, Prince Consort Road, London SW7 2BZ, United Kingdom*

[*]To whom correspondence may be addressed to: sihai.luo@ntnu.no, john.demello@ntnu.no

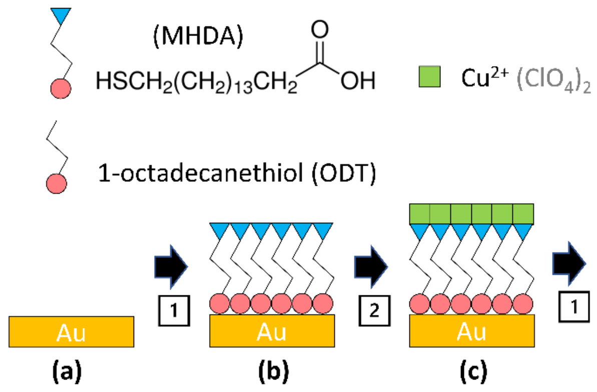

Figure S1 – The self-assembled multilayers are formed by alternately immersing a gold-coated substrate **(a)** in ethanolic solutions of MHDA (Process 1) and copper perchlorate (Process 2), rinsing thoroughly in clean ethanol between each step. The first application of Process 1 yields a densely packed monolayer of MHDA **(b)**, while the first application of Process 2 yields a layer of Cu(II) ions on top of the MHDA **(c)** that serves as an atomically thin linker on which a second thiol SAM may be attached. Repeating the two process steps adds another SAM to the assembly **(d, e)**, increasing the layer thickness by approximately 2 nm. In the final step the substrate is immersed in an ethanolic solution of octadecane thiol (Process 3), yielding an alkyl-capped upper layer **(f)**. (Note, schematic not drawn to scale).

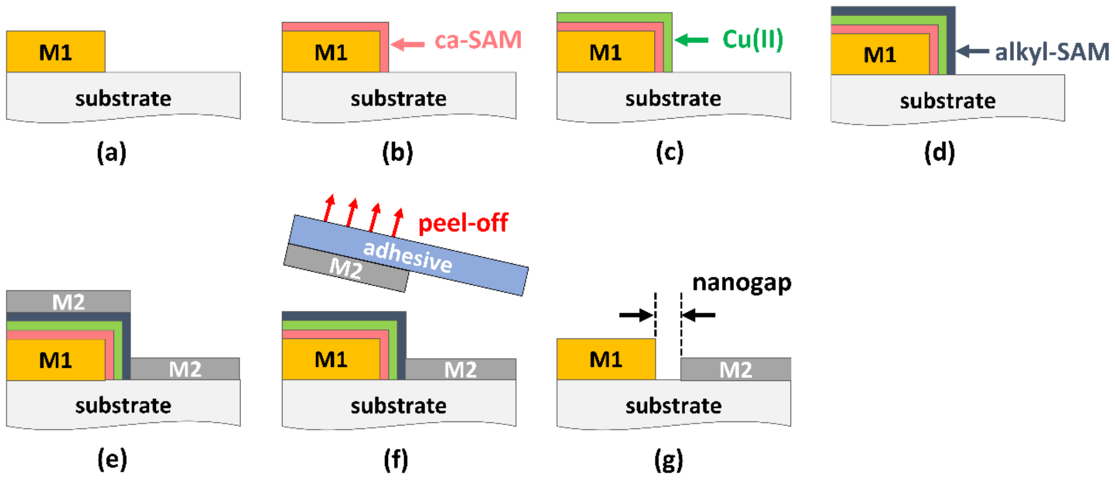

Figure S2 – Schematic showing the key processing steps for adhesion lithography with variable length molecular spacers. First, metal M1 is deposited on a substrate and patterned as required **(a)**. Second, M1 is selectively coated with a carboxylic acid-functionalised metalophilic self-assembled monolayer (ca-SAM) **(b)**. Third, the substrate is immersed in a solution of copper perchlorate, causing an atomically thin linker layer of copper ions to attach to the (outwardly facing) carboxylic acid groups on the ca-SAM molecules **(c)**. The second and third-steps may be repeated as required to add further layers to the multilayer. Fourth, for the last layer in the assembly, the multilayer is capped with an alkyl-SAM **(d)**. Fifth, metal M2 is deposited uniformly over the full area of the substrate at a substantially lower thickness than M1 **(e)**. Sixth, an adhesive film is applied to the surface of M2 and peeled away from the substrate, selectively removing M2 from those regions located directly above the multilayer **(f)**. Lastly, the multilayer is removed by UV/ozone or oxygen-plasma treatment, leaving M1 and M2 sitting in a complementary arrangement side-by-side on the substrate **(g)**, separated in the limiting case by the length of the multilayer. (Note, schematic not drawn to scale).

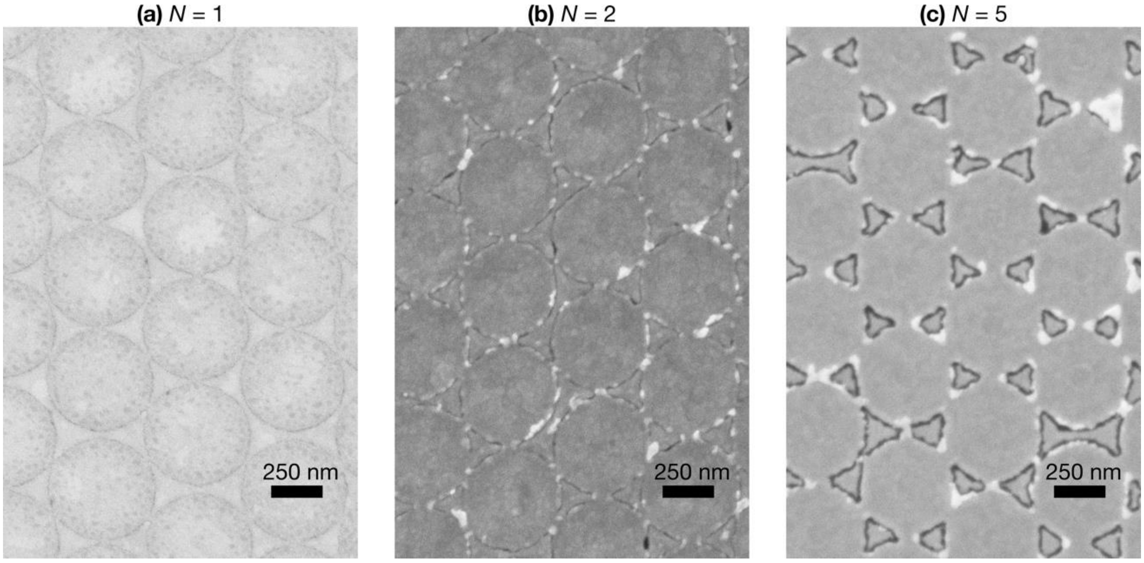

Figure S3 – SEM images of Au/Au TNG arrays, obtained using 500-nm diameter polystyrene spheres and molecular spacers of length *N* = 1 (a), *N* = 2 (b) and *N* = 5 (c).

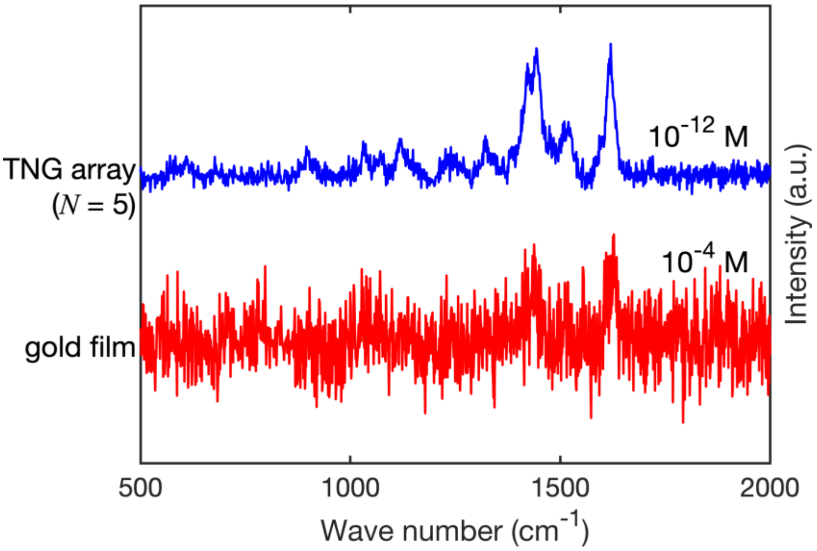

Figure S4 – Determination of the Raman scattering enhancement factor relative to a thin gold film. The blue trace shows a Raman scattering spectrum for methylene blue drop-cast from a 10^{-12} M solution onto an *N* = 5 Au/Au TNG array, fabricated using 500-nm-diameter polystyrene nanospheres under 785 nm illumination. The red trace shows a Raman scattering spectrum for MB drop-cast from a 10^{-4} M solution onto a 30-nm gold film under equivalent conditions. 10^{-12} M and 10^{-4} M represent the Raman scattering detection limits for the *N* = 5 TNG array and the gold film, respectively. Spectra were obtained using identical acquisition parameters, see Experimental section. Following Ref. S1, the analytical enhancement factor γ at 1615 cm^{-1} was determined using:

$$\gamma = \frac{I_{\mathrm{RSN}}(1615\ \mathrm{cm}^{-1})/10^{-12}}{I_{\mathrm{Au}}(1615\ \mathrm{cm}^{-1})/10^{-4}} = \frac{0.99/10^{-12}}{0.81/10^{-4}} \approx 1 \times 10^{8}.$$

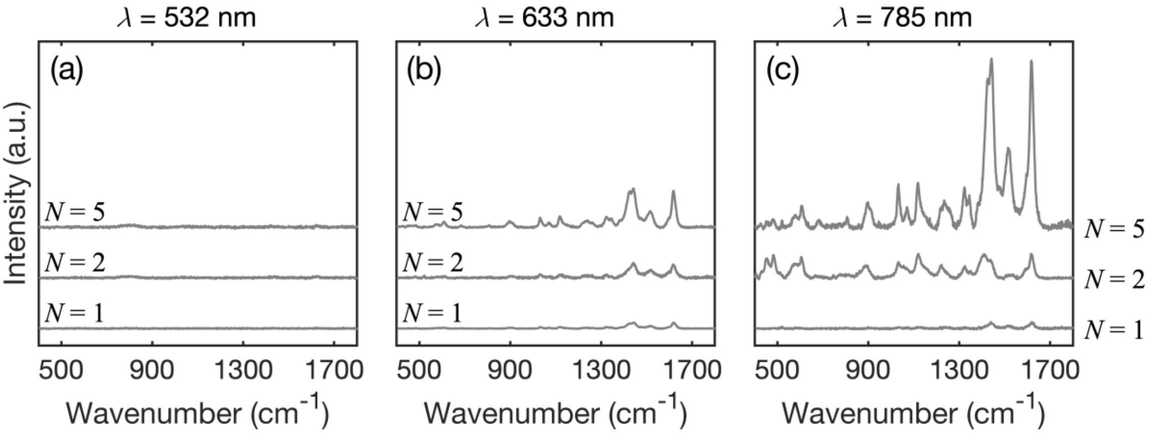

Figure S5 – Wavelength dependence of SERS signal. Raman scattering spectra for methylene blue drop-cast from a 10^{-4} M solution onto *N* = 1, 2 and 5 Au/Au TNG arrays under illumination wavelengths of 532 nm **(a)**, 633 nm **(b)** and 785 nm **(c)**. Spectra were acquired under a fixed power of 0.5 mW.

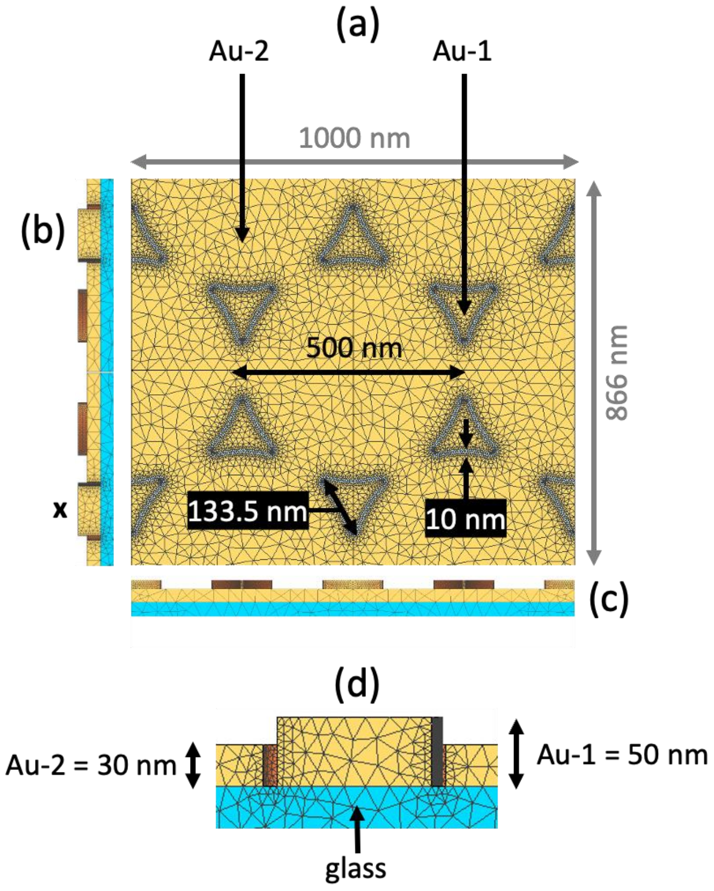

Figure S6 – Annotated top and side-view plots of geometry used for electromagnetic simulations of the TNG arrays for the illustrative case of *N* = 5. The pitch of the repeating motif was set to 500 nm (*i.e.* equal to the diameter of the polystyrene nanospheres used experimentally), the gap-width was set to 10 nm, the height of Au-1 was set to 50 nm and the height of Au-2 was set to 30 nm. No Ti adhesion layer was included between the glass substrate and the gold layers. The approximate side-length of each triangle was 133.5 nm. The geometry was the same for the cases *N* = 1 and *N* = 2, except respective gap-widths of 3 and 5 nm were set. **(a)** plan view of section of TNG array; **(b,c)** side views of the TNG array; **(d)** cross-section of triangular nanogap, corresponding to the location marked **x** in (b).

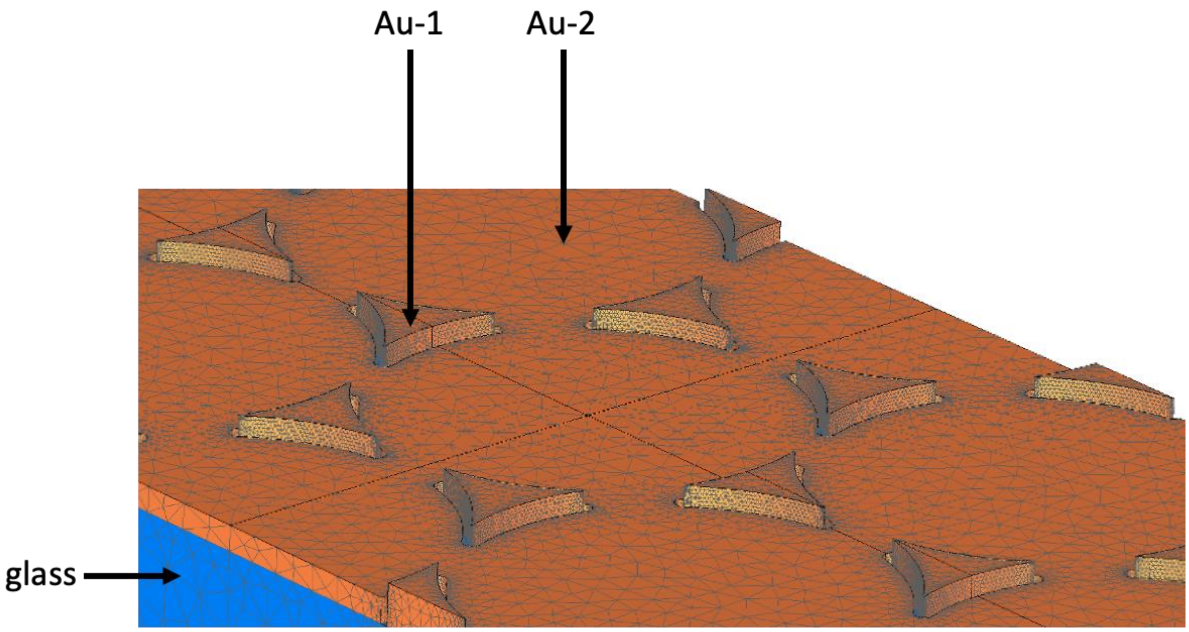

Figure S7 – Perspective view of geometry used for electromagnetic simulations for the illustrative case *N* = 5. See Figure S6 for dimensions.

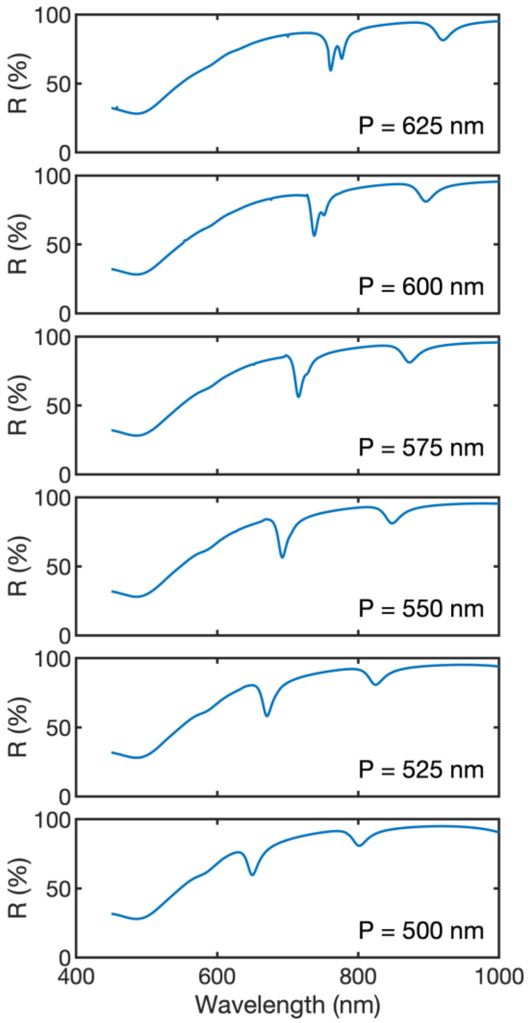

Figure S8 – **Simulated reflectance spectra for *N* = 5 Au/Au TNG arrays of varying pitch P, using the fabrication parameters shown in Figures S6 and S7.**

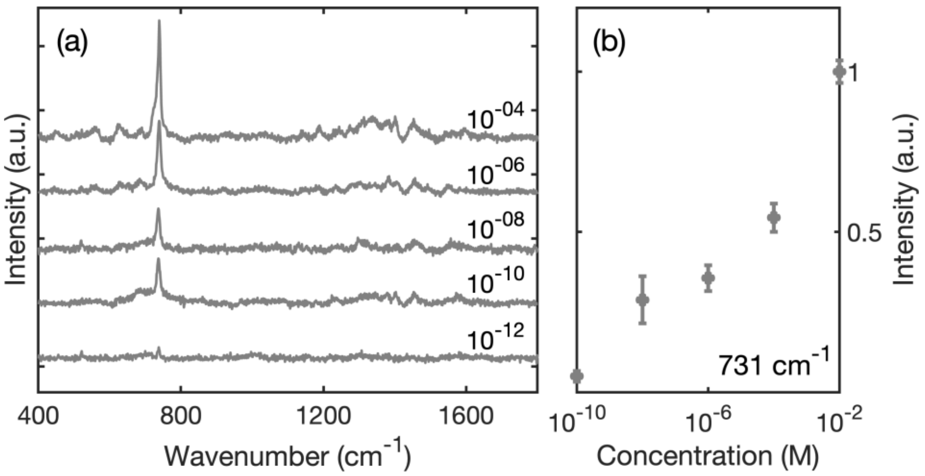

Figure S9 – Surface-enhanced Raman scattering spectra of adenine molecules on *N* = 5 Au/Au TNG arrays. (a) Experimentally determined Raman scattering spectra for adenine drop-cast onto *N* = 5 Au/Au TNG arrays from solutions of varying concentration. Spectra were obtained under equivalent conditions with a 785 nm excitation wavelength. **(b)** Plot of experimentally determined scattering intensity at 731 cm^{-1} versus dye concentration.

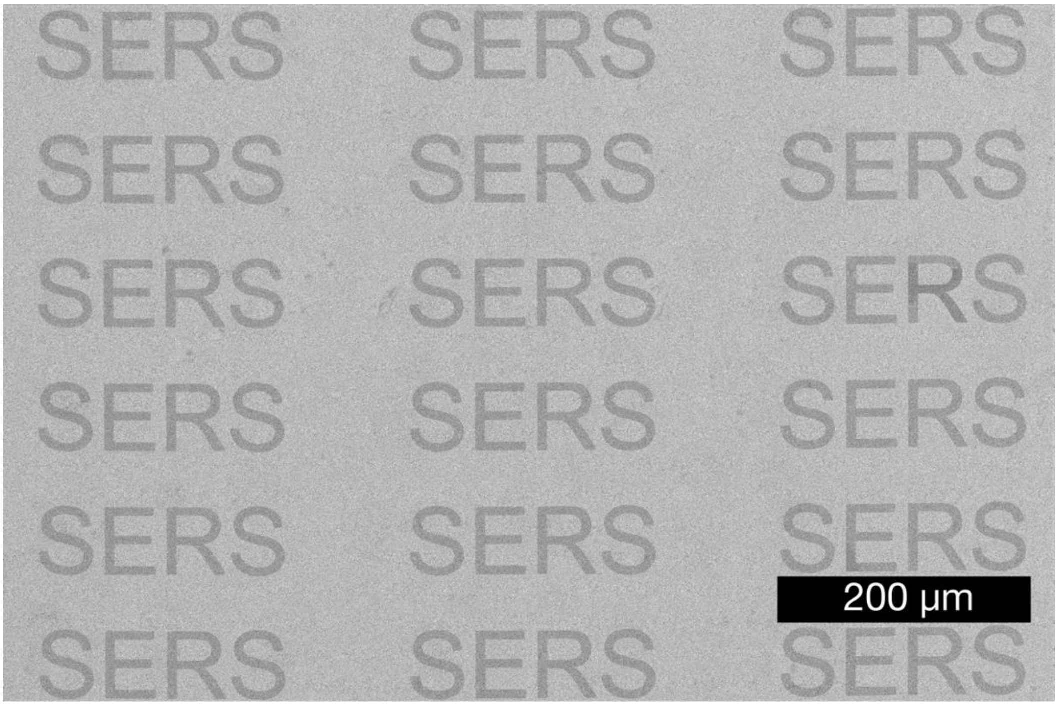

Figure S10 – Multi-scale patterning of Au/Ag nanogap arrays. The bright regions correspond to gold and the dark regions to silver. The preparation procedure was the same as for the *N* = 2 Au/Au TNG arrays, except Au-1 was photolithographically patterned with the SERS wording prior to attachment of the molecular spacers. Next, a 30 nm layer of Ag was evaporated onto the entire substrate, and the peeling procedure was then carried out in the usual way. The lithographic patterning was carried out as follows: (a) ma-N 404 negative photoresist was deposited onto the substrate by spin-coating at 2000 rpm for 5 s and 4000 rpm for 35 s; (b) the resist was lightly baked at 115 °C for 1 min; (c) it was then selectively exposed at 405 nm (200 mJ cm^{-2}) via direct writing laser lithography (Heidelberg MLA 150); (d) the substrate was immersed in ma-D 332S developer (AZ electronics materials) for 90 s, rinsed and dried; (e) Au was deposited using an e-beam evaporator across the entire glass substrate and then immersed in acetone to lift-off overnight.

Table S1 – Raman scattering enhancement factors and detection limits for MB on various SERS substrates

Fabrication method	Structure	Characteristics	Reference
**Photochemical reduction**	Ag aggregates (nanoparticles)	EF*: -<br>Detection limit: 10^{-13} M	[2]
**Recrystallization-induced self-assembly**	Cu_2O cube-like "superstructures"	EF: 8 x 10^{5}<br>Detection limit: 10^{-9} M	[3]
**Interfacial self-assembly**	Ag nanocubes on poly(dimethylsiloxane)	EF: 3.4 x 10^{6}<br>Detection limit: 10^{-10} M	[4]
**Hydrothermal synthesis and sputtering**	Au-coated ZnO nanorods	EF: -<br>Detection limit: 10^{-12} M	[5]
**Chemical synthesis**	Organic semiconductor DFP-4T films	EF: >10^{5}<br>Detection limit: 10^{-9} M	[6]
**Rapid thermal processing of thin film gold and graphene**	Au nanoparticles/graphene/Au nanoparticles	EF: -<br>Detection limit: 10^{-10} M	[7]
**Chemical synthesis**	Ag on reduced-graphene oxide	EF: 4.6 x 10^{5}<br>Detection limit: 10^{-8} M	[8]
**Self-assembly of nanoparticles**	Liquid marbles based on Ag nano-cubes	EF: 5 x 10^{8}<br>Detection limit: 10^{-16} M	[9]
**Sputtering**	Ag nano-islands on PTFE film	EF: 10^{7}<br>Detection limit: 10^{-10} M	[10]
**Nanosphere lithography, adhesion lithography**	Au triangular nanogap arrays	EF: 1.0 x 10^{8}<br>Detection limit: 10^{-12} M	This work

*EF = Enhancement Factor

References

(1) Le Ru, E. C.; Blackie, E.; Meyer, M.; Etchegoin, P. G. Surface Enhanced Raman Scattering Enhancement Factors: A Comprehensive Study. *J. Phys. Chem. C* **2007**, *111* (37), 13794–13803.

(2) Yan, W.; Yang, L.; Chen, J.; Wu, Y.; Wang, P.; Li, Z. In Situ Two-Step Photoreduced SERS Materials for On-Chip Single-Molecule Spectroscopy with High Reproducibility. *Adv. Mater.* **2017**, *29* (36), 1–7.

(3) Lin, J.; Shang, Y.; Li, X.; Yu, J.; Wang, X.; Guo, L. Ultrasensitive SERS Detection by Defect Engineering on Single Cu2O Superstructure Particle. *Adv. Mater.* **2017**, *29* (5).

(4) Li, L.; Chin, W. S. Rapid Fabrication of a Flexible and Transparent Ag Nanocubes@PDMS Film as a SERS Substrate with High Performance. *ACS Appl. Mater. Interfaces* **2020**, *12* (33), 37538–37548.

(5) Sinha, G.; Depero, L. E.; Alessandri, I. Recyclable SERS Substrates Based on Au-Coated ZnO Nanorods. *ACS Appl. Mater. Interfaces* **2011**, *3* (7), 2557–2563.

(6) Demirel, G.; Gieseking, R. L. M.; Ozdemir, R.; Kahmann, S.; Loi, M. A.; Schatz, G. C.; Facchetti, A.; Usta, H. Molecular Engineering of Organic Semiconductors Enables Noble Metal-Comparable SERS Enhancement and Sensitivity. *Nat. Commun.* **2019**, *10* (1), 1–9.

(7) Zhao, Y.; Li, X.; Du, Y.; Chen, G.; Qu, Y.; Jiang, J.; Zhu, Y. Strong Light-Matter Interactions in Sub-Nanometer Gaps Defined by Monolayer Graphene: Toward Highly Sensitive SERS Substrates. *Nanoscale* **2014**, *6* (19), 11112–11120.

(8) Chettri, P.; Vendamani, V. S.; Tripathi, A.; Singh, M. K.; Pathak, A. P.; Tiwari, A. Green Synthesis of Silver Nanoparticle-Reduced Graphene Oxide Using Psidium Guajava and Its Application in SERS for the Detection of Methylene Blue. *Appl. Surf. Sci.* **2017**, *406*, 312–318.

(9) Lee, H. K.; Lee, Y. H.; Phang, I. Y.; Wei, J.; Miao, Y. E.; Liu, T.; Ling, X. Y. Plasmonic Liquid Marbles: A Miniature Substrate-Less SERS Platform for Quantitative and Multiplex Ultratrace Molecular Detection. *Angew. Chemie - Int. Ed.* **2014**, *53* (20), 5054–5058.

(10) Šubr, M.; Petr, M.; Kylián, O.; Kratochvíl, J.; Procházka, M. Large-Scale Ag Nanoislands Stabilized by a Magnetron-Sputtered Polytetrafluoroethylene Film as Substrates for Highly Sensitive and Reproducible Surface-Enhanced Raman Scattering (SERS). *J. Mater. Chem. C* **2015**, *3* (43), 11478–11485.
